# Associations between Late Lactate Clearance and Clinical Outcomes in Adults with Hyperlactataemia in the Setting of Diabetic Ketoacidosis

**DOI:** 10.3390/jcm13164933

**Published:** 2024-08-21

**Authors:** Aashish Kumar, Christopher Anstey, Ra’eesa Doola, Philippa Mcllroy, Stephen Whebell, Kiran Shekar, Antony Attokaran, Prashanti Marella, Kyle White, Stephen Luke, Alexis Tabah, Kevin Laupland, Mahesh Ramanan

**Affiliations:** 1 Intensive Care Unit, Logan Hospital, Brisbane, QLD 4131, Australia; aashish.kumar@health.qld.gov.au; 2 School of Medicine and Dentistry, Griffith University, Sunshine Coast, QLD 4575, Australia; c.anstey@griffith.edu.au; 3 Department of Nutrition and Dietetics, Princess Alexandra Hospital, Brisbane, QLD 4102, Australia; raeesa.doola@health.qld.gov.au; 4 Princess Alexandra Southside Clinical Unit, School of Clinical Medicine, The University of Queensland, Brisbane, QLD 4072, Australia; kyle.white@health.qld.gov.au; 5 Intensive Care Unit, Cairns Hospital, Cairns, QLD 4870, Australia; philippa.mcilroy@health.qld.gov.au; 6 Intensive Care Unit, Townsville University Hospital, Townsville, QLD 4814, Australia; stephen.whebell@health.qld.gov.au; 7 Adult Intensive Care Services, The Prince Charles Hospital, Chermside, QLD 4032, Australia; kiran.shekar@health.qld.gov.au; 8 Faculty of Health, Queensland University of Technology (QUT), Brisbane, QLD 4000, Australia; prashanti.marella@health.qld.gov.au (P.M.); alexis.tabah@health.qld.gov.au (A.T.); kevin.laupland@qut.edu.au (K.L.); 9 Faculty of Medicine, University of Queensland, Brisbane, QLD 4072, Australia; antony.attokaran@health.qld.gov.au; 10 Intensive Care Unit, Rockhampton Hospital, Rockhampton, QLD 4700, Australia; 11 Intensive Care Unit, Caboolture Hospital, Brisbane, QLD 4510, Australia; 12 Intensive Care Unit, Queen Elizabeth II Jubilee Hospital, Brisbane, QLD 4108, Australia; 13 Intensive Care Services, Mackay Base Hospital, Mackay, QLD 4740, Australia; stephen.luke2@health.qld.gov.au; 14 College of Medicine and Dentistry, James Cook University, Townsville, QLD 4811, Australia; 15 Intensive Care Unit, Redcliffe Hospital, Brisbane, QLD 4020, Australia; 16 Intensive Care Unit, Royal Brisbane and Women’s Hospital, Brisbane, QLD 4006, Australia; 17 Critical Care Division, The George Institute for Global Health, University of New South Wales, Sydney, NSW 2000, Australia

**Keywords:** diabetic ketoacidosis, lactate, critical care, diabetes

## Abstract

**Objective**: This study aimed to determine the associations between lactate clearance in hyperlactataemic patients with diabetic ketoacidosis (DKA) and intensive care unit (ICU), hospital length of stay (LOS), and case-fatality. **Methods**: A retrospective, multicentre, cohort study of adult patients admitted to ICU with hyperlactataemia and a primary diagnosis of DKA from twelve sites in Queensland, Australia was conducted utilising pre-existing datasets that were linked for research purposes. The patients were divided into early and late lactate clearance groups; the early lactate clearance group included patients whose lactate returned to <2.0 mmol/L within 12 h, and the remainder were classified as late lactate clearance group. **Results**: The final dataset included 511 patients, 427 in the early lactate clearance group and 84 in the late lactate clearance group. Late lactate clearance was associated with increasing ICU LOS (β = +15.82, 95% CI +0.05 to +31.59, *p* < 0.049), increasing hospital LOS (β = +7.24, 95% CI +0.11 to 14.37, *p* = 0.048) and increasing Acute Physiology and Chronic Health Evaluation(APACHE) III score (ICU LOS outcome variable β = +1.05, 95% CI +0.88 to +1.22, *p* < 0.001; hospital LOS outcome variable β = +3.40, 95% CI +2.22 to 4.57, *p* < 0.001). Hospital case-fatality was not significantly different (2.2% in the early clearance group vs. 1.7% in the late clearance group, *p* = 0.496). **Conclusions**: In hyperlactataemic patients with DKA, late lactate clearance was associated with a statistically significant increase in both ICU and hospital LOS, though the clinical significance in both is minor.

## 1. Introduction

Diabetic ketoacidosis (DKA) is a condition that poses significant healthcare burden, impacting nearly 8 in 1000 patients with diabetes annually, with a mortality between 1 and 5% [1,2]. DKA arises due to deficiency or absence of circulating insulin, leading to hyperglycaemia, elevated ketones, and a high anion-gap metabolic acidosis [2,3].

Previous studies have demonstrated associations between hyperlactataemia and increased in-hospital case-fatality [4,5]. However, production and clearance of lactate is a dynamic process that necessitates evaluation over the time course of the disease [6,7]. In disease states such as sepsis, burns and myocardial disease, lactate clearance is an independent predictor of mortality [8]. The aetiology of hyperlactataemia in these settings is largely attributed to anaerobic glycolysis [4,9,10]. Conversely, the aetiology of hyperlactataemia in the setting of DKA is theorised to be due to alternate mechanisms, such as thiamine deficiency, which is commonly observed in diabetes, and the association between lactate clearance and clinical outcomes in DKA remains undefined [7,10,11,12,13].

The aim of this study was to determine associations between lactate clearance in hyperlactataemic patients with DKA and clinical outcomes, including intensive care unit (ICU) length of stay (LOS), hospital LOS, and ICU and hospital case-fatality.

## 2. Materials and Methods

The authors conducted a retrospective, multicentre cohort study that utilised data collected for quality assurance purposes by the Australia and New Zealand Intensive Care Society (ANZICS), Centre for Outcome and Resource Evaluation (CORE), and Adult Patient Database (APD) linked with data from eCritical MetaVision™ (iMDsoft, Boston, MA, USA) from twelve discrete intensive care units in Queensland, Australia. Data collected included patient demographics, hemodynamic parameters, outcome data, biochemistry, observations, and medications.

The approval for this study, with waiver of consent, was granted by the Metro South Human Research Ethics Committee (HREC/2022/QMS/82024) and data access was approved by the ANZICS CORE Directorate.

Data were collected pertaining to all eligible patients from 1 January 2015 to 31 December 2021. The inclusion criteria were adult patients (Age 18 and older) admitted to ICU with a primary diagnosis of DKA and lactate ≥2.0 mmol/L. This included patients with Type 1, Type 2, undifferentiated or other (for example, post-pancreatectomy) types of diabetes. Patients with applied limitations of life-sustaining care at the time of ICU admission, including “not for resuscitation”, “not for intubation”, “not for vasoactive drugs”, or “not for dialysis” orders, were excluded due to their known associations with worse outcomes to avoid confounding [14].

### Data Management and Statistical Analysis

The variables extracted included demographic information, admission diagnosis, physiological variables used in Acute Physiology and Chronic Health Evaluation III (APACHE-III) scoring, lactate, Australia and New Zealand Risk of Death (ANZROD), biochemistry, observations, medications, vital status at hospital discharge, and lengths of stay in ICU and hospital.

The cohort was divided into two groups based on time to clearance of lactate with hyperlactataemia defined as a serum lactate concentration ≥2.0 mmol/L, as has been previously defined in the published literature [4,5,7,8,12]. The ‘early lactate clearance group’ consisted of patients who returned to lactate <2.0 mmol/L within 12 h of ICU admission; the remainder were classified as the ‘late lactate clearance group’, based on the methodology described by Morgan et al. [12].

The primary outcome was ICU LOS. The secondary outcomes included hospital LOS, need for vasopressors, receipt of mechanical ventilation, and the requirement for renal replacement therapy.

Statistical analysis was performed using STATA version 17.0. Continuous data were assessed for normality using the Shapiro–Wilk test and subsequently summarised as mean and standard deviation (if normally distributed) or median and interquartile range (if not normally distributed). Categorical data were summarised as proportions and percentages. The two groups were compared using the Mann–Whitney U test and the Pearson chi-squared test, respectively, for continuous and categorical variables.

The data formed a longitudinal time-series (panel data), and general multivariable modelling was undertaken with the patient ID set as the panel variable and number of hours to lactate clearance as the temporal variable.

The primary outcome, ICU LOS, was evaluated using mixed-effects multivariable logistic regression, with results presented as Beta (β), the regression coefficient slope, with 95% confidence interval (CI). The primary exposure variable was the lactate clearance group. Site was entered into the model as a random effect, with patients nested within sites. Fixed effects were sequentially added to generate a fully saturated model, from which non-significant covariates were removed to yield a final, adjusted model.

Non-linear modelling was accomplished using a general exponential washout curve. Statistical analysis was performed using STATA version 17.0 with the level of significance set at α < 0.05.

## 3. Results

Over the study period, 858 ICU admissions with DKA were recorded at the participating centres. Of these, 511 ICU admissions were hyperlactatemic on admission, of whom 84 patients met the criteria for late lactate clearance, with the remaining 427 patients being classified as early lactate clearance. The average age in the early lactate clearance group was 53 years (34–61) and 49 years (30–59) in the late lactate clearance group. In the early lactate clearance group, 219 patients (51.3%) were male, compared to 27 patients (32.5%) in the late lactate clearance group. Nine case-fatalities occurred, three in the ICU and six after ICU discharge. Univariate analysis of demographic data is summarised in Table 1.

Initial arterial blood gas (ABG) results within four hours of admission to ICU are summarised in Table 2. Patients in the late lactate clearance group had a mean lactate concentration of 5.3 mmol/L (IQR 4.0–6.7 mmol/L) within the first four hours, compared to 2.8 mmol/L (IQR 2.2–3.5 mmol/L; *p* < 0.001) in the early lactate clearance group. The late lactate clearance group also had a higher anion gap of 22 mmol/L (IQR 15–28 mmol/L) as compared to 8 mmol/L (IQR 13–23 mmol/L; *p* < 0.001) in the early lactate clearance group. There was no significant difference between the initial blood glucose concentrations: 26 mmol/L (IQR 14 to 37 mmol/L) in the late clearance group as opposed to 25 mmol/L (IQR 14 to 36 mmol/L; *p* = 0.867) in the early lactate clearance group. A positive association was also noted between early lactate clearance and lower admission whole blood sodium concentration with 136 mmol/L (IQR 133–139 mmol/L) in the early lactate clearance group vs. 139 mmol/L (IQR 133–145 mmol/L) in the late lactate clearance group, *p* = 0.041.

### Outcomes

The results of univariate analysis of primary and secondary outcomes are summarised in Table 3. Late lactate clearance was associated with a mean ICU LOS of 59 h (95% CI 40–99 h) vs. 48 h (95% CI 32–76 h; *p* < 0.001) in the early lactate clearance group and a mean hospital LOS of 141 h (95% CI 83–300 h) in the late lactate clearance group vs. 110 h (95% CI 69–195 h; *p* < 0.001) in the early lactate clearance group. Late lactate clearance was also significantly associated with a lower proportion of male patients, higher APACHE-II, APACHE-III, and ANZROD scores.

Patients in the late lactate clearance group had an increased need for vasopressors (43.1%) compared to the early lactate clearance group (13.8%; *p* < 0.001). A positive association with the proportion of invasive positive pressure ventilation was also noted with late lactate clearance (22.4% late lactate clearance vs. 7.9% early lactate clearance; *p* = 0.034). The proportionate use of NIV was also significantly higher in the late lactate clearance group (6.9% late lactate clearance vs. 1.9% early lactate clearance; *p* = 0.045). No significant associations were noted regarding the requirement for renal replacement therapy or overall ventilation hours with both IPPV and NIV.

The results of multivariable modelling are presented in Table 4. Patients in the late lactate clearance group had a significantly longer ICU LOS compared to the early lactate clearance group (β = +15.8, 95% CI +0.05 to +31.59, *p* = 0.049). An association with increased ICU LOS was also noted for patients with high APACHE-III scores (β = +1.05, 95% CI +0.88 to +1.22, *p* < 0.001), with the median APACHE-III score for patients in the late lactate clearance group being 77 (IQR 60 to 96) compared to 59 (IQR 47 to 76) in the early lactate clearance group. The model building details are outlined in Appendix A (Table A1).

Multivariable modelling with hospital LOS as the outcome variable also demonstrated a significantly longer LOS in the late lactate clearance group as opposed to the early lactate clearance group (β = +7.24, 95% CI +0.11 to +14.37, *p* = 0.048), and an association of higher APACHE-III scores with late lactate clearance (β = +3.40, 95% CI +2.22 to +4.57, *p* < 0.001) was found. The model building details are outlined in Appendix A (Table A2).

**Figure 1:**
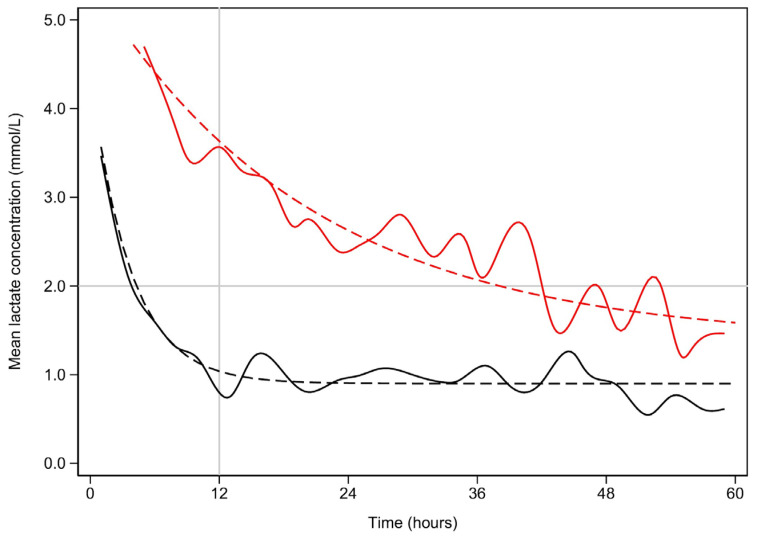
Mean lactate concentration (mmol/L) vs. time (hours) for early and late lactate clearance.

**Figure A1:**
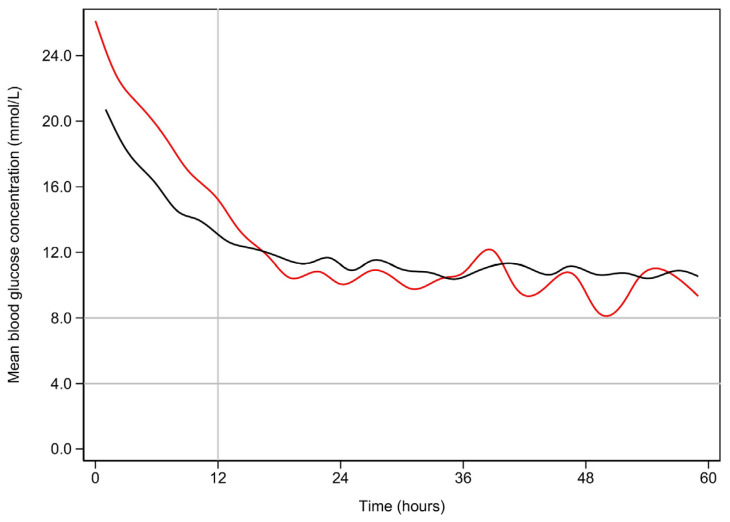
Mean blood glucose (mmol/L) vs. time (hours) for early and late resolution of lactate.

**Figure A2:**
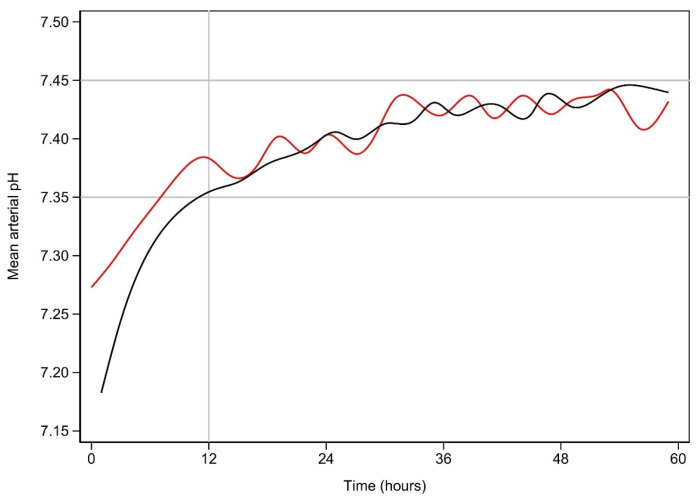
Mean arterial pH vs. time (hours) for early and late resolution of lactate.

**Figure A3:**
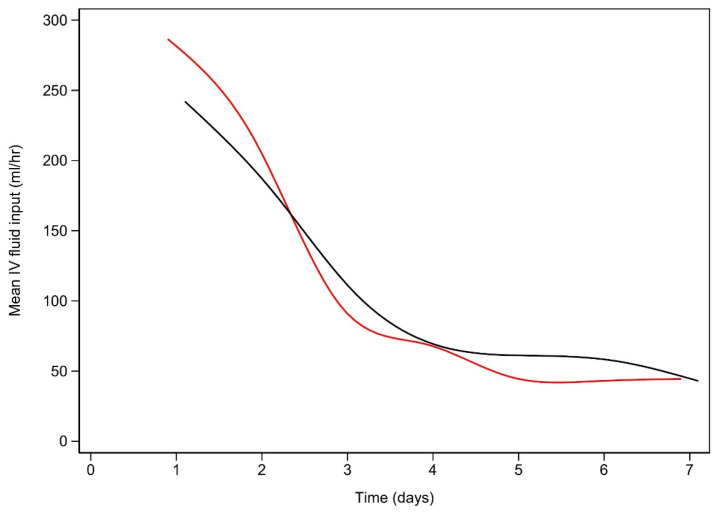
Intravenous crystalloid (mL/h) use vs. time (days) for early and late lactate clearance.

**Figure A4:**
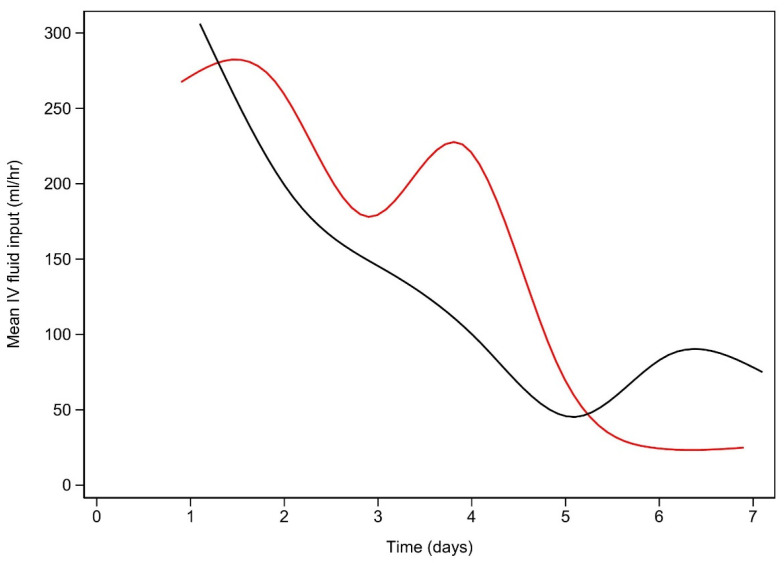
Intravenous colloid (mL/h) use vs. time (days).

Figure 1 demonstrates the clearance of lactate over time for the first 60 h among the early and late lactate clearance groups. Figure A1 and Figure A2 (see Appendix A) demonstrate the time course for glucose and pH for both clearance groups. Despite the notable difference in lactate clearance over time, both groups demonstrated similar trajectories for resolution of glucose and pH. Figure A3 and Figure A4 (see Appendix A) demonstrate the mL/h of crystalloid and colloid received, demonstrating that both groups received similar volumes of crystalloid; however, the late lactate clearance group received higher volumes of colloid.

Early lactate resolution is plotted in black, while late lactate resolution is plotted in red. The solid lines represent the best fit splines. The dashed lines represent the non-linear fitted plots. The upper bound is marked by the horizontal line. For the sake of clarity, lactate concentrations above 5 mmol/L are not shown. Similarly, the time scale is limited to 60 h.

Half lives:

Early—t_1/2_ = 2.6 h (95% CI 2.3–2.9, *p* < 0.001)

Late—t_1/2_ = 14.1 h (95%CI 12.2–17.1, *p*< 0.001)

The difference is also significant (*p* < 0.001).

## 4. Discussion

In this large cohort study, late lactate clearance among patients admitted to ICU with DKA was independently associated with a longer ICU LOS. The other major factor affecting ICU length of stay was increasing APACHE-III score. Mixed-effects modelling including significant univariate variables demonstrated that late clearance of lactate was statistically associated with a marginally longer ICU LOS, but not likely to be clinically significant. Mortality between the groups was not significantly different.

Multiple factors could account for these findings. The emerging literature suggests that hyperlactataemia in the setting of DKA may have separate aetiologies and ambiguous clinical significance compared to lactate elevations seen in other areas of critical care, such as sepsis, myocardial infarction, burns or trauma [3,8,9,12,15]. The physiologic mechanism of hyperlactataemia in the latter situations is primarily attributed to anaerobic metabolism secondary to tissue hypoxia [16]. Late clearance of lactate in these circumstances is independently associated with worse clinical outcomes, including ICU and hospital case-fatality, and ICU and hospital LOS [3,13]. However, in the setting of DKA, the aetiology of and clinical significance of hyperlactataemia and the significance of its clearance remains an area of debate, though its contribution to overall acidemia in DKA is likely to increase ICU and hospital LOS [4,7,8,10,12,13,17,18].

Factors beyond anaerobic glycolysis could account for the accumulation of lactate in DKA, including a catecholaminergic state, severe dehydration, metformin use, and thiamine deficiency [1,7,9,19]. This is suggested by our results where it was noted that despite the difference between lactate clearance over time between the two groups, biochemical resolution of pH and glucose was similar, suggesting that the hyperlactataemia observed was due to a process separate from the DKA. Derangement in overall metabolic profile is a significant influencer of ICU and hospital LOS in DKA, and interventions specifically targeting lactate aetiology may lead to a faster improvement [12].

A potential therapeutic option for further exploration is the administration of thiamine to hyperlactataemic patients with DKA. Thiamine deficiency is frequent among diabetic patients due to insulin deficiency causing impaired enteral thiamine absorption and renal thiamine reuptake, with thiamine deficiency being a known precipitant of hyperlactataemia [10,11]. Administration of thiamine may hasten lactate clearance, with faster resolution of metabolic profile and decreased ICU and hospital LOS [1,7,12]. This requires evaluation in prospective, interventional studies.

Our study demonstrated that patients with higher APACHE-III scores and late lactate clearance had a higher ICU LOS compared to those who had early clearance of lactate. Multiple factors could account for this finding. Diabetes often co-exists with significant comorbidities, including obesity, cardiac disease, and metabolic syndrome, which influence illness severity scores [20,21]. Furthermore, diabetic patients are at increased risk for sepsis, which is a common trigger for DKA, and is associated with high morbidity and mortality, which is also associated with high illness severity scores [8,20,22,23,24,25,26]. Sepsis may be a contributor to hyperlactataemia in patients with DKA, alongside alternate biochemical mechanisms [24].

The relationship between lactate clearance and case-fatality remains uncertain, investigated in only two small studies. Ibrahim et al. [6] performed a retrospective observational study of 107 patients, with a 7.54% case-fatality rate and demonstrated an association between lactate clearance and decreasing thirty-day case-fatality. Similarly, in a cohort of 40 patients, Taskin et al. [13] found decreasing lactate levels to be associated with improving case-fatality. Larger studies are required to explore this potential association.

The strengths of this study are that it is the largest study to evaluate the impact of lactate clearance on ICU LOS and other clinical outcomes in patients with DKA and has analysed data from large registries that have been utilised for research purposes. However, this study is limited by its retrospective observational design, potential for unknown confounders, and missing data. The numbers of patients in the early and late lactate clearance groups were disproportionate, affecting the robustness of the conclusions of the study. A notable limitation is the lack of data on the type of diabetes the patient had and the specific trigger for DKA, which can impact the aetiologies of the raised lactate, as patients whose DKA is triggered by sepsis, or acute coronary syndromes can be predicted to have a higher lactate level. Similarly, data regarding patients’ medications such as metformin or antipsychotics, the duration since the diagnosis of diabetes, and their diet were not available, all of which can impact lactate production and clearance.

## 5. Conclusions

Patients presenting with DKA frequently demonstrate a concomitant hyperlactataemia, which has an unclear aetiology and may be associated with poorer clinical outcomes. In this study, late lactate clearance was associated with increased ICU and hospital LOS. Future studies should be aimed at reviewing interventions that may expedite lactate clearance and potentially improve clinical outcomes for this group of patients.

## Figures and Tables

**Table 1:** Patient baseline characteristics.

Variable	Early Lactate Clearance ^1^ n = 427	Late Lactate Clearance ^1^ n = 84	*p*-Value ^2^
Age _(years)_	53 (34, 61)	49 (30, 59)	0.301
Male sex	219 (51.3%)	27 (32.5%)	0.011
Comorbidities			
Respiratory	6 (1.41%)	2 (2.41%)	0.501
Cardiovascular	5 (1.17%)	1 (1.20%)	0.981
Renal	17 (3.98%)	1 (1.20%)	0.207
IDDM	367 (85.9%)	68 (81.0%)	0.975
Pressure injury	8 (1.87%)	1 (1.82%)	0.925
Risk of Death Scores			
APACHE-II Score	21 (16, 25)	25 (18, 28)	0.545
APACHE-III Score	59 (47, 76)	77 (60, 96)	0.139
APACHE-III Risk of death _(%)_	0.038 (0.019, 0.057)	0.043 (0.018, 0.068)	0.176
ANZROD _(%)_	0.008 (0.003, 0.013)	0.011 (0.004, 0.018)	0.381

^1^ Median (IQR) or frequency (%); ^2^ Mann–Whitney U test; Pearson Chi-squared test. Abbreviations: ICU = intensive care unit; IDDM = insulin-dependent diabetes; APACHE = Acute Physiology and Chronic Health Evaluation; ANZROD = Australia and New Zealand Risk of Death.

**Table 2:** Arterial blood gas (ABG) results within four hours of admission to ICU.

Variable	Early Lactate Clearance N = 427	Late Lactate Clearance N = 84 ^1^	*p*-Value ^2^
Lactate _(mmol/L)_	2.8 (2.2, 3.5)	5.3 (4.0, 6.7)	<0.001
Electrolytes			
Sodium (Na^+^) _(mmol/L)_	136 (133, 139)	139 (133, 145)	0.041
Potassium (K^+^) _(mmol/L)_	4.1 (3.5, 4.5)	4.0 (3.5, 4.4)	0.974
Chloride _(mmol/L)_	107 (103, 113)	109 (105, 113)	0.138
Ionised calcium _(mmol/L)_	1.20 (1.13, 1.24)	1.19 (1.14, 1.26)	0.539
Anion gap _(mmol/L)_	18 (13, 23)	22 (15, 28)	0.032
Blood glucose _(mmol/L)_	25 (14, 36)	26 (14, 37)	0.867
Creatinine _(µmol/L)_	95 (65, 123)	115 (50, 260)	0.062
Acid-base			
pH	7.26 (7.15, 7.32)	7.23 (7.10, 7.31)	0.663
paCO_2 (mmHg)_	21 (14, 27)	20 (15, 25)	0.937
paO_2 (mmHg)_	104 (78, 123)	116 (95, 138)	0.798
Bicarbonate _(mmol/L)_	11 (6, 15)	7 (4, 13)	0.065
Base Excess _(mmol/L)_	−13 (−20, −6)	−15 (−22, −11)	0.102
Oximetry			
Haemoglobin _(g/L)_	112 (98, 126)	115 (99, 128)	0.851
Oxyhaemoglobin _(%)_	96 (93, 97)	96 (95, 97)	0.935
Carboxyhaemoglobin _(%)_	0.8 (0.3, 1.3)	0.8 (0.3, 1.2)	0.946
Methaemoglobin _(%)_	0.5 (0.3, 1.0)	0.5 (0.3, 1.2)	0.829

^1^ Median (IQR) or frequency (%); ^2^ Mann–Whitney U Test; Pearson Chi-squared test.

**Table 3:** Univariate analysis of primary and secondary outcomes.

Variable	Early Lactate Clearance N = 427 ^1^	Late Lactate Clearance N = 84 ^1^	*p*-Value ^2^
Length of stay (hours)			
ICU	53 (34, 61)	49 (30, 59)	0.301
Hospital	219 (51.3%)	27 (32.5%)	0.011
IPPV	34 (7.9%)	13 (22.4%)	0.034
NIV	7 (1.6%)	4 (6.9%)	0.079
Vasopressors	59 (13.8%)	25 (43.1%)	<0.001
RRT	14 (3.3%)	4 (6.9%)	0.497
Ventilation hours			
IPPV	68 (30, 137)	73 (42, 171)	0.736
NIV	5 (1, 8)	11 (6, 11)	0.002

^1^ Median (IQR) or Frequency (%); ^2^ Mann–Whitney U Test; Pearson Chi-squared test. Abbreviations: IPPV = invasive positive pressure ventilation; NIV = non-invasive ventilation; RRT = renal replacement therapy.

**Table 4:** Multivariable mixed-effects model after sequential deletion of non-significant predictors with ICU and hospital LOS as the outcome variables.

Variable	β (95% CI)	*p*-Value
ICU Length of Stay		
Late lactate clearance	+15.82 (+0.05, +31.59)	0.049
APACHE-III Score	+1.05 (+0.88, +1.22)	<0.001
Hospital Length of Stay		
Late lactate clearance	+7.24 (+0.11, +14.37)	0.048
APACHE-III Score	+3.40 (+2.22, +4.57)	<0.001

Abbreviations: APACHE = Acute Physiology and Chronic Health Evaluation.

## Data Availability

Data cannot be shared publicly due to institutional ethics, privacy, and confidentiality regulations. Data released for research under Sect. 280 of the Public Health Act 2005 require an application to the Director General of Queensland Health (PHA@health.qld.gov.au).

## Appendix A

**Table A1:** Multivariable analysis using a fully saturated model with ICU LOS as the outcome variable.

Predictor	β (95% CI)	*p*-Value
Early lactate clearance	+18.23 (+1.03, +35.43)	0.039
Age (years)	−0.12 (−0.36, +0.13)	0.355
Female sex	+7.52 (−0.27, +15.31)	0.059
Co-morbidities		
Renal	+0.16 (−37.44, +37.77)	0.993
Respiratory	−31.29 (−65.05, +2.47)	0.062
Cardiovascular	+5.64 (−21.06, +32.35)	0.679
Frailty score (referenced to 1)		
2	−9.74 (−37.85, +18.37)	0.497
3	−23.63 (−52.31, +5.06)	0.107
4	−27.34 (−58.24, +3.56)	0.096
5	−31.99 (−68.02, + 4.05)	0.102
6	−24.09 (−55.04, +6.87)	0.127
7	−38.74 (−86.14, +8.66)	0.134
8	−58.01 (−145.77, +29.76)	0.195
BMI (kg/m^2^)	+0.07 (−0.62, +0.77)	0.839
APACHE-III Score	+0.70 (+0.52, +0.87)	<0.001

Abbreviations: BMI = body mass index; APACHE = Acute Physiology and Chronic Health Evaluation.

**Table A2:** Multivariable analysis using a fully saturated model with hospital LOS as the outcome variable.

Predictor	β (95% CI)	*p*-Value
Early lactate resolution	+8.23 (+0.03, +16.43)	0.049
Age (years)	+1.01 (−0.89, +1.13)	0.332
Female sex	+13.38 (−12.97, +39.35)	0.713
Co-morbidities		
Renal	−0.48 (−2.69, +1.74)	0.681
Respiratory	−14.18 (−25.62, +2.73)	0.064
Cardiovascular	+10.16 (−8.76, +29.08)	0.293
Frailty score (referenced to 1)		
2	+3.29 (−59.35, +65.95)	0.918
3	+25.39 (−38.11, +88.90)	0.433
4	+9.60 (−57.62, +76.81)	0.780
5	+82.99 (−6.93, +172.91)	0.070
6	+60.16 (−23.45, +143.77)	0.158
7	+13.69 (−2.09, +25.29)	0.212
8	+11.49 (−252.61, +275.58)	0.932
BMI (kg/m^2^)	+0.62 (−1.47, +2.71)	0.561
APACHE-III Score	+1.78 (+1.13, +2.42)	<0.001

Abbreviations: BMI = body mass index; APACHE = Acute Physiology and Chronic Health Evaluation.

**Table A3:** Univariate analysis of number of comorbidities.

Number of Comorbidities	Early Lactate Clearance N = 427	Late Lactate Clearance N = 84 ^1^	*p*-Value ^2^
0	119 (27.9%)	17 (20.2%)	0.423
1	281 (65.8%)	62 (73.8%)	
2	15 (3.5%)	5 (6.0%)	
3	9 (2.1%)	0	
4	3 (0.7%)	0	
5	0	0	

^1^ Median (IQR) or frequency (%); ^2^ ANOVA.

Early lactate resolution is plotted in black, while late lactate resolution is plotted in red. The normal fasting range is marked by the horizontal lines.

Early lactate resolution is plotted in black, while late lactate resolution is plotted in red. The normal range is marked by the horizontal lines.

Early lactate resolution is plotted in black, while late lactate resolution is plotted in red.

Usage is calculated at the end of each day. Early lactate resolution is plotted in black, while late lactate resolution is plotted in red.
